# Monostotic fibrous dysplasia at C7 treated with vertebroplasty: a case report and review of the literature

**DOI:** 10.1186/s12957-019-1717-2

**Published:** 2019-11-09

**Authors:** Xin Xin, Jianhong Feng, Chen Yue, Tao Jin, Xinxin Liu

**Affiliations:** 1 Department of Orthopaedics, Ankang Center Hospital, Ankang, 725000 Shaanxi Province People’s Republic of China; 2 Department of Magnetic Resonance Imaging, Hong Hui Hospital of Xi’an Jiaotong University, Xi’an, 710054 Shaanxi Province People’s Republic of China

**Keywords:** Fibrous dysplasia, Vertebroplasty, Cervical vertebra, Spine

## Abstract

**Background:**

Monostotic fibrous dysplasia (MFD) involving the spine is rare, and the treatment options are controversial. Surgery is needed when patients suffer from persistent pain, spinal cord compression/injury, and vertebral collapse/instability. Treatment methods include biopsy/observation, corpectomy with instrumented fusion, posterior fusion, vertebroplasty (VP), curettage and bone graft, and complete removal of the vertebra with a combined anterior and posterior fusion procedure.

**Case presentation:**

The patient was a 56-year-old woman with a 2-year history of neck pain. No obvious abnormalities were detected on neurological or physical examination, and laboratory findings were all within normal limits. An imaging examination suggested a C7 vertebral bone tumor. The patient refused to continue conservative observation treatment and requested surgery. Open VP of the C7 vertebral body was carried out, and her postoperative neck pain was completely relieved. The postoperative pathological results supported the diagnosis of fibrous dysplasia, and the patient was ultimately diagnosed with MFD. At the 12-month follow-up visit, the patient reported no clinical symptoms, and no signs of tumor recurrence were detected.

**Conclusion:**

VP can relieve pain while stabilizing the spine. Thus, the surgical treatment of MFD vertebral lesions by VP is a valuable option.

## Background

Fibrous dysplasia (FD) was first reported by Lichtenstein in 1938 [1]. FD is a benign bone tumor in which normal bone tissue and bone marrow are replaced by proliferative fibrous tissue [2], representing approximately 5–7% of benign bone tumors [3]. FD can be classified as monostotic fibrous dysplasia (MFD) or polyostotic fibrous dysplasia (PFD). PFD may be accompanied by endocrine disorders such as McCune-Albright syndrome (MAS) [4]. MFD accounts for 70% of the reported cases of FD. However, MFD involving the cervical vertebrae is quite rare, and the treatment methods are controversial. Surgery is needed when patients suffer from persistent pain, spinal cord compression/injury, and vertebral collapse/instability. Here, we report a case of MFD at C7 that was successfully treated with vertebroplasty (VP). We also searched PubMed and the Web of Science with the keywords “fibrous dysplasia” and “spine” to evaluate the literature regarding the surgical treatment of cervical MFD and have provided a summary of the treatment of spinal FD with VP.

## Case presentation

**Fig. 1:**
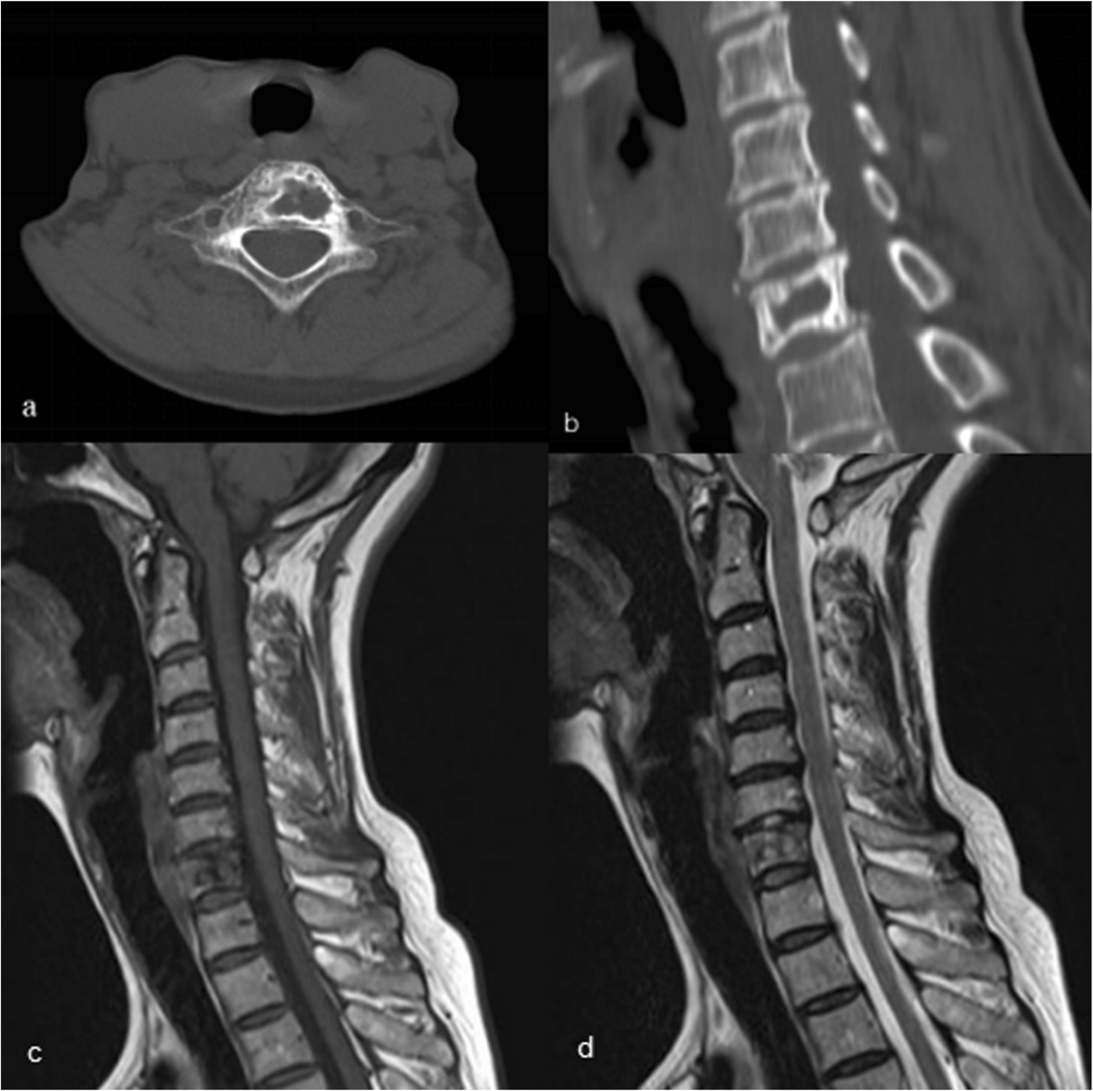
Preoperative imaging of the patient with cervical fibrous dysplasia. **a**, **b** CT images demonstrated low density of the seventh cervical vertebra, with high-density hardening visible around the edges. **c**, **d** MRI scans of the cervical spine indicated an expansile lytic lesion with isointensity on T1-weighted imaging and hyperintensity on T2-weighted imaging

**Fig. 2:**
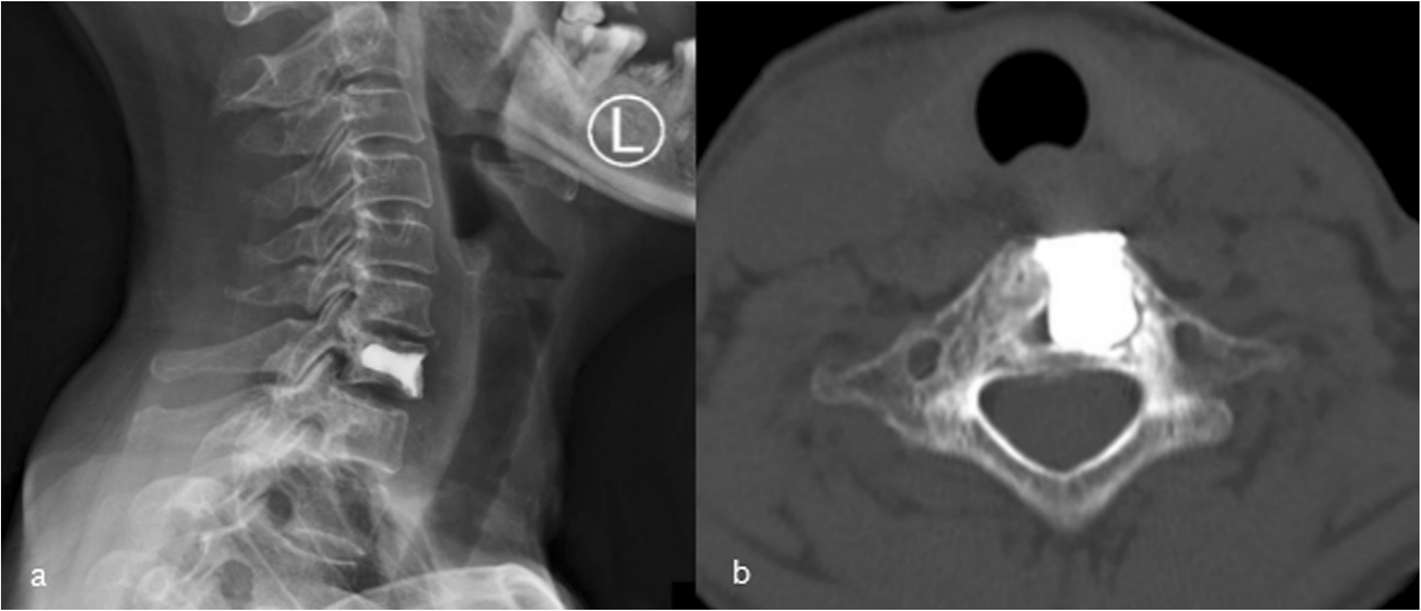
Three days after surgery imaging of the C7 vertebral body after cement filling (**a**, **b**): **a** X-ray, **b** CT

**Fig. 3:**
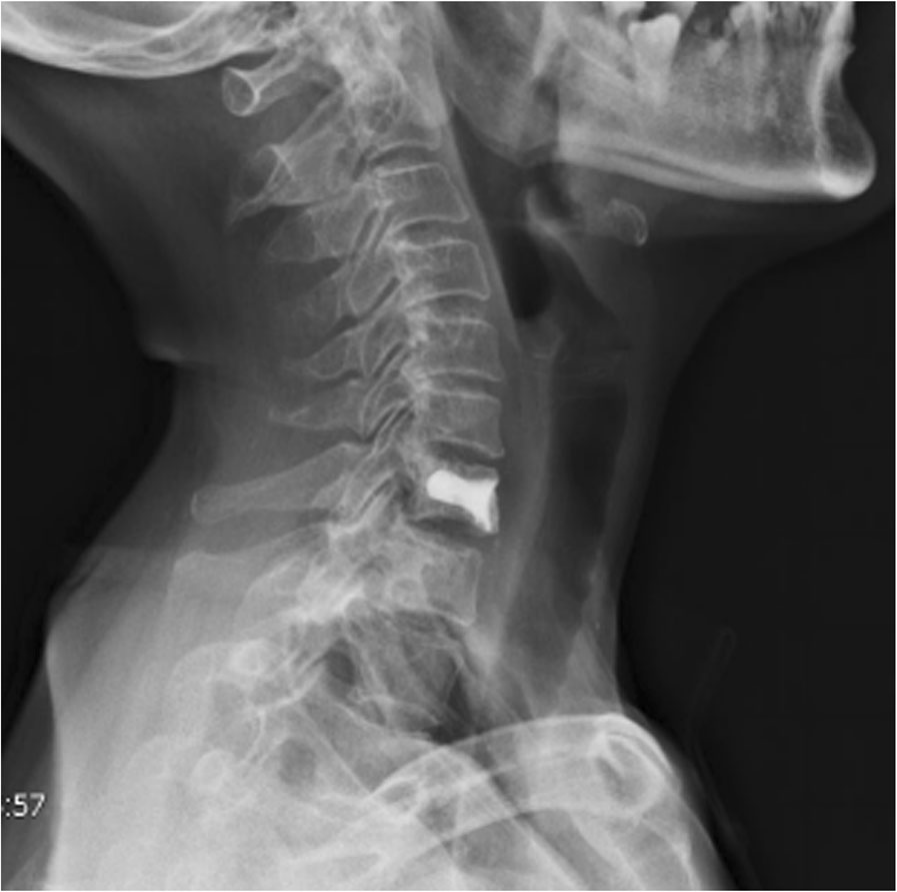
Six months after surgery imaging of X-ray

A 56-year-old female patient was admitted to our hospital with a 2-year history of neck pain. No obvious abnormalities were detected on neurological or physical examination, and laboratory findings were all within normal limits. Computed tomography (CT) (Fig. 1a, b) demonstrated low density in the seventh cervical vertebra, with high-density hardening visible around the edges. Magnetic resonance imaging (MRI) of the cervical spine (Fig. 1c, d) indicated an expansile lytic lesion with isointensity on T1-weighted imaging and hyperintensity on T2-weighted imaging. These findings were explained to the patient as the possible causes of neck pain, and options for continued conservative observation or surgical treatment were provided. The patient refused to continue conservative observation treatment and requested surgery. The preoperative treatment team communicated sufficiently about the case, considered the existing clinical data of benign bone tumors, and recommended two surgical treatment options: (1) open biopsy with direct excision and internal fixation, which would involve extensive trauma and a long recovery time, or (2) open biopsy with bone cement injection, with later treatment options to be determined according to the pathological results after surgery and reoperation to remove the lesion, if necessary. The patient chose the scheme 2. C7 VP was performed after inducing general anesthesia. Imaging examinations were performed at 3 days, 6 months, and 1 year after surgery (Fig. 2a, b; 3a; 4a, b). The postoperative pathological results supported the diagnosis of FD (Fig. 5a, b), and the patient was ultimately diagnosed with MFD. At the 12-month follow-up visit, the patient reported no clinical symptoms, and no signs of tumor recurrence were detected.

**Fig. 4:**
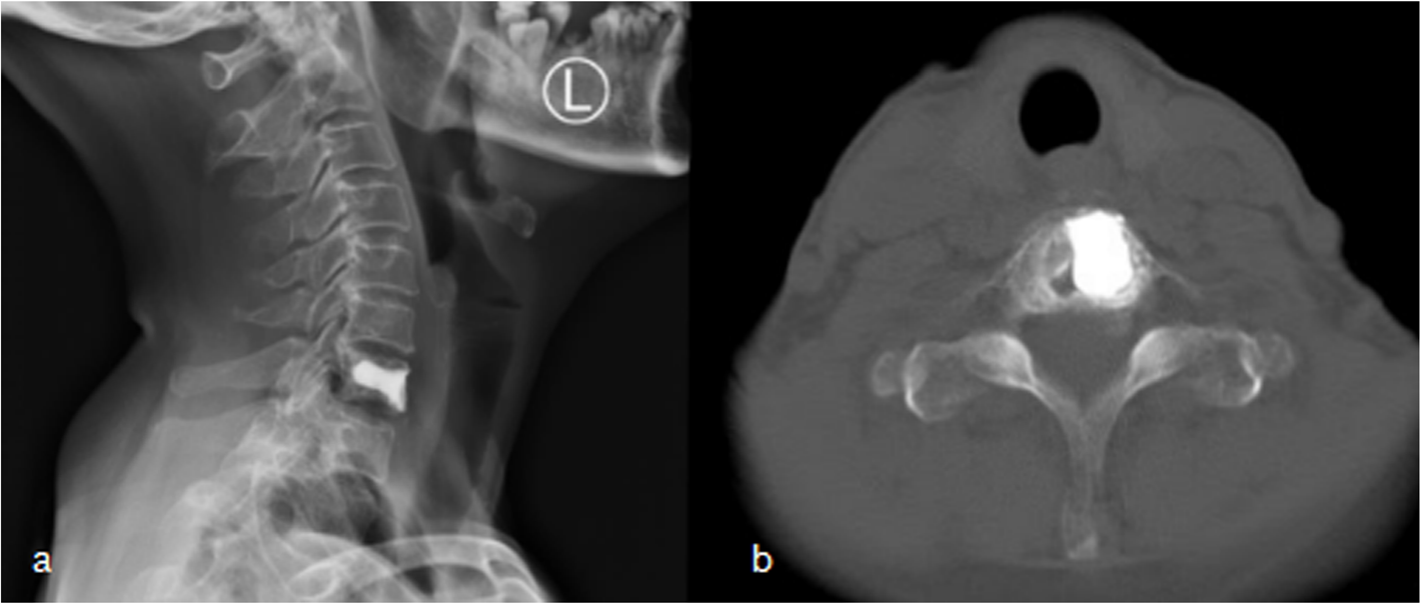
Twelve months after surgery imaging of X-ray (**a**) and CT (**b**)

**Fig. 5:**
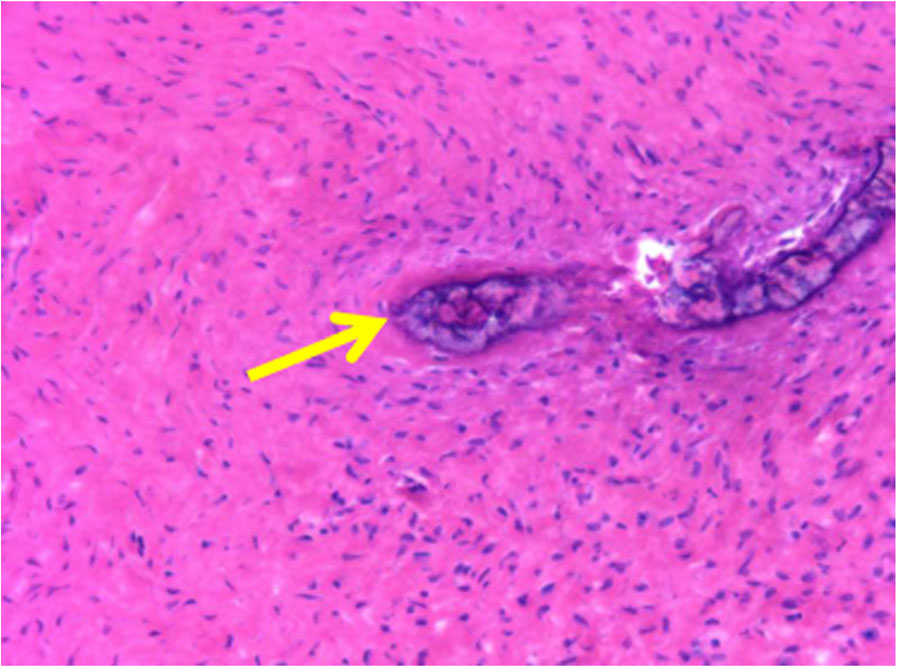
Pathological HE staining × 10 tumor-like hyperplastic fibers arranged sparsely in a woven pattern with fibrous bone (yellow arrow)

## Discussion

FD is a non-hereditary disease; one possible mechanism is the multi-synthetase-activating mutation of the GNAS1 gene on chromosome 20q13.2-13.3 in somatic cells during embryonic development [5, 6]. Because the number of mutant cells often decreases with age, FD has age-related self-limiting characteristics [7] and a high incidence rate among teenagers in the general population. Lesions most often present in the long bones of the legs, arms, ribs, and pelvis, as well as in craniofacial bones. The spine is involved in 1.4–5.5% of FD lesions [8, 9]. FD progresses slowly, and malignancies rarely occur [10]. The natural courses of MFD, PFD, and MAS are quite different [2]. MFD generally stops developing once skeletal development ceases, and the lesion itself can be repaired. PFD and MAS can continue to develop after the bones mature, and the lesions can continue to progress, resulting in new pathological fractures or deformities [9, 11]. FD without local symptoms does not require surgical treatment [2], but the possibility of pathological fracture and deformity should be carefully assessed. Surgery is needed when patients suffer from persistent pain, spinal cord compression/injury, and vertebral collapse/instability.

Our literature review revealed 17 cases of surgically treated MFD involving cervical vertebrae, and the characteristics of these cases (including the one case presented in this study) are summarized in Table 1 [12–24]. None of these patients had pathological fractures or neurological deficits prior to surgery, and good results were achieved after surgical treatment. For lesions located on the posterior side of the vertebral body, laminectomy and fusion with or without internal fixation were performed [13, 14, 16, 18, 19, 21–23]. Three treatments were performed for vertebral body invasion: (1) Curettage of the lesion in the vertebral body followed by bone grafting was used in some cases [12, 15], although lesion recurrence and bone graft resorption were reported after this treatment in a long-term literature review [25, 26]. (2) Corpectomy with instrumented fusion was also used as a treatment and is a relatively thorough method for removing the lesion [16, 17, 23, 24]. If there is no spinal cord compression from the vertebral body, if the tumor is benign, and if the progression is slow, overly aggressive treatment should be avoided [21], especially because these tumors are rarely malignant. (3) VP [20] was used for the treatment of osteoporotic vertebral collapse, vertebral angiomas, and malignant tumors and has achieved good clinical results [27, 28]. VP has the advantages of less trauma, a shorter hospitalization duration, and faster recovery.

**Table 1:** Clinical characteristics of surgical treatment of cervical MFD

Report	Sex	Age	Site	Symptoms	Treatment	Outcome (months)
Rosendahl-Jensen (1956) [12]	F	35	C4	Post-traumatic	Curettage and bone grafting	Asymptomatic (12)
Stirrat et al. (1989) [13]	M	25	C2	Neck pain	Posterior occipital-C4 fusion, P	Asymptomatic (24)
Hu et al. (1990) [14]	M	41	C2	Neck pain	Arthrodesis at C1-C3, P	Asymptomatic (18)
Ohki (1990) [15]	F	20	C2	No pain, local expansion	Curettage and bone grafting	Asymptomatic (60)
Villas and Martínez-Peric (1992) [16]	M	11	C4	Painful torticollis	Removal with instrumented fusion, A/P	Asymptomatic (48)
Marshman et al. (2004) [17]	M	35	C3	Pathological fracture	Corpectomy with instrumented fusion, A	Asymptomatic (18)
Arantes et al. (2008) [18]	F	53	C1	Neck pain	Laminectomy with curettage, P	Asymptomatic (48)
Sambasivan et al. (2008) [19]	M	35	C4	Neck pain	Laminectomy, P	Asymptomatic (3 weeks)
Kotil and Ozyuvaci (2010) [20]	M	55	C2	Neck pain	VP, A	Asymptomatic (12)
Meredith and Healey (2011) [21]	M	41	C2	Neck pain	C1-C3 fusion, P	Asymptomatic (240)
Bangash et al. (2011) [22]	M	16	C1	Headache	Laminectomy, P	Asymptomatic (18)
Wu et al. (2013) [23]	M (1)	37	C4	Neck pain	Excision, A/P	Asymptomatic (24)
	M (2)	48	C2-C3	Incidental finding	Curettage, P	Asymptomatic (34)
	M (3)	53	C2	Neck pain	Curettage, P	Asymptomatic (33)
Yang et al. (2016) [24]	M	21	C7	Neck pain	Corpectomy with instrumented fusion, A	Asymptomatic (36)
	F	42	C7	Neck pain	Corpectomy with instrumented fusion, A	Asymptomatic (6)
XIN 2019	F	56	C7	Neck pain	VP, A	Asymptomatic (12)

*A* anterior approach, *F* female, *M* male, *P* posterior approach, *VP* vertebroplasty, *PVP* percutaneous vertebroplasty

Our literature review revealed only 7 patients (including the 1 patient reported in this study) who underwent VP for the treatment of spinal fibrous dysplasia, as shown in Table 2 [20, 23, 29–31]; 2 patients had MFD, and 5 had PFD. Pathological fractures occurred in 5 patients, involving 13 vertebrae. The outcomes of these patients have been uniformly good, and no complications related to cement implantation have been reported. During the follow-up period, the patients experienced pain relief and showed no deformity or limited progression of spinal deformity.

**Table 2:** Reported details of spinal FD treated by VP/PVP/KP

Reports	Sex	Age	Location	Type of FD	Pathological fracture(s)	Symptoms	Treatment	Outcome (months)
Deen and Fox (2005) [29]	F	25	T8-L2	Polyostotic	T8, L2	Back pain	T8, T9, T10, L2, PKP, P	Asymptomatic (6)
Dang et al. (2007) [30]	M	35	C2, C3, C6	Polyostotic	C2, C3	Neck pain	C2, C3, PVP	Asymptomatic (12)
Kotil and Ozyuvaci (2010) [20]	M	55	C2	Monostotic		Neck pain	C2, PVP, A	Asymptomatic (12)
Chen et al. (2011) [31]	F	56	T8, T9, T10	Polyostotic	T8, T9, T10	Back pain	T9, T10, PKP, P	Asymptomatic (12)
Wu et al. (2013) [23]	M (4)	32	T5–11	Polyostotic	T6, T7, T10	Back pain	VP, P	Asymptomatic (28)
	F (5)	38	C2, C4, C6, T2-T6, T8, T12	Polyostotic	T2, T4, T5	Back pain, paraparesis	Laminectomy, VP (T2-T8), P	Asymptomatic (24)
Xin	F	56	C7	Monostotic		Neck pain	C7, VP, A	Asymptomatic (12)

*A* anterior approach, *F* female, *M* male, *P* posterior approach, *VP* vertebroplasty, *PVP* percutaneous vertebroplasty, *PKP* percutaneous kyphoplasty

The injection of bone cement increases the strength of the vertebral body and effectively improves the stability of the spinal system [32]. The chemical activity of the cement during the solidification process generates heat, destroying the nerve endings around the fracture, resulting in an analgesic effect [33]. Poly methyl methacrylate (PMMA) bone cement itself has a certain cytotoxic effect, which may lead to decreased cell metabolic activity, cell death or injury, the growth of new tumor cells, and decreased cell proliferation in vertebral bodies that have been solidified using cement [34, 35].

There are several challenges in the application of VP for MFD: (1) It is difficult to accurately diagnose the disease preoperatively because there is a lack of characteristic imaging manifestations, and the FD diagnosis depends on pathology results; in addition, CT-guided percutaneous biopsy for the evaluation of spinal lesions has a reported diagnostic accuracy of approximately 90% [36]. However, the positive FD diagnosis rate is low [23, 37]. Because it is impossible to make a clear diagnosis before surgery, other methods may be applied; thus, it is recommended that open biopsy be carried out when vertebral FD is suspected [23, 36]. (2) Expansive osteolysis and pathological fractures in FD may lead to the leakage of bone cement upon injection. The edge of vertebral sclerosis in FD can reduce cement leakage. VP has been suggested to reduce the cement leakage caused by vertebral fracture during kyphoplasty (KP) stretching [23]. (3) The efficacy of VP treatment is uncertain due to the lack of large-scale case studies and long-term follow-up results. With the increase in the application of VP for the treatment of vertebral tumors, the efficacy of VP in terms of pain relief and spinal stabilization and the underlying mechanisms will be further demonstrated.

## Conclusion

VP has been widely used for the treatment of bone tumors, but its uses for the treatment of spinal FD is relatively rare. To the best of our knowledge, this report represents the second case in which VP is used for the treatment of spinal MFD. MFD is a benign tumor with slow progression. As VP can relieve pain while stabilizing the spine, the surgical treatment of MFD vertebral lesions by VP is a valuable option.

## Data Availability

The data are available from the corresponding author on reasonable request.
